# Looking at Both Sides: Integrating Data From Both Hemispheres Is Crucial in Rodent Neuroscience

**DOI:** 10.1111/ejn.70249

**Published:** 2025-09-06

**Authors:** Annakarina Mundorf, Sebastian Ocklenburg

**Affiliations:** ^1^ Medical School Hamburg Institute for Systems Medicine and Department of Human Medicine Hamburg Germany; ^2^ Medical School Hamburg ICAN Institute for Cognitive and Affective Neuroscience Hamburg Germany; ^3^ Department of Psychology MSH Medical School Hamburg Hamburg Germany; ^4^ Biopsychology, Institute of Cognitive Neuroscience, Faculty of Psychology Ruhr University Bochum Bochum Germany

**Keywords:** hemispheric asymmetry, histology, lateralization, neuroimaging techniques, standardized protocols

## Abstract

Despite the overwhelming prevalence of rodent‐based research in neuroscience, with over 8700 studies published until March 2025 in the *European Journal of Neuroscience* alone (based on a targeted PubMed search using rodent‐related keywords), one striking reality stands out: only a handful—24 studies—have explicitly addressed hemispheric asymmetries (using the same search with asymmetry‐related terms). While this number is not exhaustive, it serves to exemplify the relatively limited focus on hemispheric lateralization in rodent studies. This notable gap in the literature highlights a pervasive underappreciation for the role of brain lateralization in rodents, a critical area of investigation that has been widely studied in human neuroscience but remains largely unexplored in animal models. In light of this, it becomes clear that a fundamental shift in research priorities is needed to unlock the full potential of rodent models in understanding brain organization and its implications for neurological and psychiatric disorders.

## Graphical abstract

Hemispheric asymmetries remain critically underexamined in rodent neuroscience, limiting our ability to fully capture brain organization and its relevance to neurological and psychiatric disorders. Despite technological advances, current practices often obscure lateralized processes due to pooled data, inconsistent dissections, and lack of hemisphere‐specific analysis. This article calls for a shift toward standardized, hemisphere‐aware methodologies, leveraging imaging, genetics, and bilateral recording, to enhance reproducibility, reveal overlooked patterns of asymmetry, and unlock the translational potential of rodent models in understanding brain lateralization.

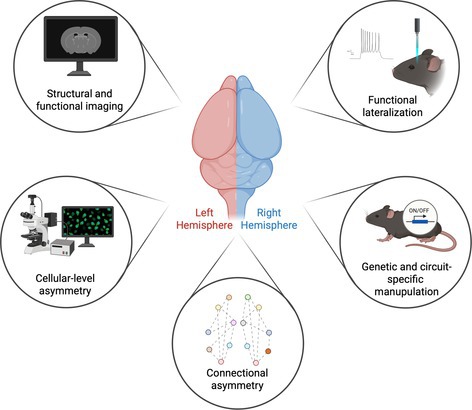

## 1 Background on Hemispheric Asymmetries in Rodent Models

Hemispheric asymmetries represent a fundamental organizational principle in the vertebrate brain (Lapraz et al. 2024). These asymmetries encompass behavioral differences, such as preferences for using one limb over the other; functional differences, including variations in neural activity or processing between hemispheres; and structural differences, which refer to anatomical variations in brain regions across the two sides (Toga and Thompson 2003; Ocklenburg and Guo 2024; Ströckens et al. 2025). Together, these levels contribute to specialization within the brain and underlie various cognitive functions. Importantly, sexual dimorphism and sex differences influence hemispheric asymmetries, with males and females differing in several key aspects. For example, some research indicates that males exhibit a greater degree of asymmetry, such as larger differences in size or activity between hemispheres, compared to females (Wisniewski 1998; Hirnstein et al. 2019). In terms of direction, both sexes often show leftward asymmetry in specific regions, but males tend to display greater rightward asymmetry in some areas, whereas females may exhibit more pronounced leftward asymmetry in others (Guadalupe et al. 2017; Nazlee et al. 2022). Behaviorally, males have been reported to show a slightly higher prevalence of left‐handedness compared to females, highlighting sex‐specific patterns in lateralized behaviors (Papadatou‐Pastou et al. 2008, 2020). In contrast, rodent studies often fail to show consistent sex differences in limb preference patterns (Stieger et al. 2021; Ströckens et al. 2025). These patterns are thought to be shaped, in part, by hormonal influences, especially sex steroids, which impact the development and function of the brain (Hausmann and Güntürkün 2000; Ocklenburg and Güntürkün 2024). Such findings underscore the necessity of considering sex as a biological variable in the study of brain asymmetry.

Building on this, large‐scale studies from the ENIGMA‐Laterality Consortium and others have underscored the significance of hemispheric asymmetries in human brain structure and function (Kong et al. 2022; Guadalupe et al. 2017; Ocklenburg et al. 2024; Ocklenburg and Guo 2024). These investigations reveal consistent patterns, such as the association between hand preference and frontoparietal tract asymmetry (Howells et al. 2018), and the predominance of the left hemisphere in language processing (Malik‐Moraleda et al. 2022), highlighting the centrality of lateralization to brain organization.

Despite growing recognition of lateralization in humans, rodent research continues to overlook key aspects of hemispheric asymmetry. For instance, although animals do not show handedness identical to humans, many species (including rodents) exhibit limb preferences, a well‐established, noninvasive, and easily quantifiable form of behavioral asymmetry (Manns et al. 2021; Ströckens et al. 2025). Yet, rodent studies often analyze data from a single hemisphere, especially under specific experimental conditions, potentially undermining the validity and reproducibility of their findings. Additionally, certain brain states, such as sleep, known to influence hemispheric function in birds, aquatic mammals, and reptiles through mechanisms like unihemispheric sleep (Rattenborg et al. 2000), remain poorly understood in rodents and may represent an overlooked factor modulating lateralization.

This oversight may obscure crucial insights into brain function, particularly in the context of neurological and psychiatric disorders. Building on the influential perspective article, “On the Other Hand: Including Left‐Handers in Cognitive Neuroscience and Neurogenetics” (Willems et al. 2014), which emphasized the need to consider lateralization in human brain studies, we advocate for a similar shift in rodent research that broadly acknowledges hemispheric asymmetries at both behavioral and neuronal levels. Limb preference, for instance, provides a straightforward and underutilized behavioral marker for such investigations.

A major challenge in advancing this line of research lies in the absence of standardized protocols for hemispheric dissection and analysis in rodents, which makes it difficult to incorporate asymmetry into routine experimental designs. While research in human participants has made significant strides in understanding hemispheric asymmetry, animal studies still lack comparable methodological breakthroughs. Emerging rodent studies have begun to investigate lateralized brain alterations across psychiatric, neurodevelopmental, and neurodegenerative disorders, many of which parallel findings in humans (Table 1). These examples underscore the importance of systematically incorporating bilateral analysis and hemisphere‐specific dissection in future research to enhance translational relevance.

**TABLE 1:** Selected rodent studies on hemispheric asymmetries across psychiatric, neurodevelopmental, and neurodegenerative disorders, with corresponding evidence in humans.

Condition	Animal model/paradigm	Hemispheric asymmetry	References	Evidence of altered asymmetry in humans (examples)
Anxiety	Fear conditioning; predator odor	↑ PKCβII in right amygdala; ↑ dopamine in right (♂) or left (♀) amygdala	Orman and Stewart 2007; Sullivan et al. 2009, 2014	Wang et al. 2018; Mundorf et al. 2021b; Ocklenburg et al. 2023
Depression	Chronic mild stress; forced swim test	↓ *Bdnf* in right frontotemporal cortex; L‐sided behavioral biases (paw preference + head turning)	Farhang et al. 2014; Soyman et al. 2018; Ecevitoglu et al. 2020	Bruder et al. 2017; Mundorf et al. 2024
Schizophrenia	Nogo‐A‐deficiency; maternal immune activation	Young adults: ↑ iNOS in left frontal + parietal cortices Old adults: bilateral ↓ NR1; altered D_2_ receptor in mPFC; lateralized turning	Krištofiková et al. 2013; Mundorf et al. 2021a	Oertel et al. 2010; Ocklenburg et al. 2013; Gutman et al. 2022; Schijven et al. 2023
Autism	Transgenic mouse models (POND dataset: Chd8, 16p11 lines)	Distinct asymmetry phenotypes; ↑ isocortex volume in wild‐type vs. transgenic; ↓ ventral striatum and retrohippocampal regions in transgenics	Rivera‐Olvera et al. 2024	Markou et al. 2017; Li et al. 2023
Chronic stress/posttraumatic stress	Chronic social or restraint stress; repetitive low‐level blast	↑ Cytogenesis in right mPFC; reduced hemispheric difference in infralimbic and prelimbic cortex; ↓ atypical dendritic length in left anterior cingulate cortex; ↑ left turns; ↑ p‐tau Thr181 right hippocampus	Czéh et al. 2007; Perez‐Cruz et al. 2007; Perez Garcia et al. 2021; Mundorf et al. 2022	Borawski et al. 2022; López‐Castro et al. 2023
Addiction	Ethanol withdrawal; nicotine injection	Right‐turning bias, altered right amygdala signaling	Carlson and Drew Stevens 2006; Bergstrom et al. 2010	Cao et al. 2023
Alzheimer's disease	3xTg‐ad; AppNL‐G‐F mouse models	Right hippocampal atrophy; asymmetric plaque deposition	Muntsant and Giménez‐Llort 2020; Sacher et al. 2020	Lubben et al. 2021; Roe et al. 2021; Lu et al. 2023

*Note:* This table provides an overview of key animal models, experimental paradigms, and observed hemispheric asymmetries reported in the literature. Studies are grouped by disorder or condition and highlight representative findings of lateralized molecular, neuroanatomical, and behavioral alterations. While this summary includes multiple examples per disorder to illustrate common patterns, it is not exhaustive. For a comprehensive review and detailed discussion of hemispheric asymmetries in rodent models of pathology, refer to Mundorf and Ocklenburg (2023).

Abbreviations: Bdnf, Brain‐derived neurotrophic factor; mPFC, medial prefrontal cortex; PKCβI, protein kinase C beta II isoform.

## 2 Relevance of Hemispheric Asymmetry to Neurological and Psychiatric Research

Regardless of the increased awareness of hemispheric asymmetry in the human brain, research in rodent neuroscience still often combines data across hemispheres or overlooks hemispheric factors in analyses (for examples, see Zhang et al. 2023; Jain et al. 2024; Kremer et al. 2024; Sayar‐Atasoy et al. 2024; Tian et al. 2024; Van Deusen et al. 2025), thereby limiting the depth of their findings. This methodological limitation is compounded by a widespread preference for simplified study designs that omit hemisphere‐specific factors to facilitate data collection. Large reference atlases like the Allen Mouse Brain Atlas lack hemisphere‐specific data, complicating the interpretation of asymmetry in rodent brains. For instance, while the Allen Mouse Atlas includes data from multiple individuals, the hemispheres often come from different subjects, or, in some cases, only one hemisphere is available (Oh et al. 2014; Eastwood et al. 2019; Wang et al. 2020), creating potential data inconsistencies and undermining the validity of asymmetry studies.

Nonetheless, evidence increasingly supports the need to integrate hemispheric analysis into rodent neuroscience. Several studies have shown that the amygdala displays lateralized differences in connectivity, function, and neurotransmitter expression across mammals (Ocklenburg et al. 2022). As the amygdala is a key structure in the clinical neuroscience of affective disorders, these asymmetries need to be considered in experiments using rodent models for affective disorders. Similarly, asymmetrical dopamine expression has been observed in the nucleus accumbens of rats (Budilin et al. 2008). Given the critical role of the nucleus accumbens in the reward circuitry and its involvement in mood disorders, schizophrenia, and substance use disorders (Chambers et al. 2001; Cao et al. 2021; Xu et al. 2024), hemispheric differences warrant attention in relevant animal experiments. Ignoring hemispheric asymmetries in reward circuitry models risks missing key neurobiological mechanisms underlying addiction and mood regulation.

Building on the need to rigorously examine hemispheric differences in rodent models, recent empirical evidence illustrates these points. An analysis of a dataset involving over 2000 mice, including models for autism spectrum disorder, revealed distinct hemispheric brain asymmetries (Rivera‐Olvera et al. 2024). Larger volumes in the right hemisphere were found in the anterior regions, while larger volumes in the left hemisphere were observed in the posterior regions, differing from human brain asymmetries (Kong et al. 2022). These findings highlight that asymmetry patterns in rodent models of neurodevelopmental disorders differ from human lateralization, warranting careful, species‐specific interpretation. Notably, autism spectrum disorder models showed lateralization patterns paralleling those seen in atypical human development, underscoring the value of rodent models while emphasizing the need to consider interspecies differences (Rivera‐Olvera et al. 2024).

Extending this work, researchers analyzed data from six neuroimaging cohorts encompassing 3500 mice to examine neuroanatomical asymmetries (Silberfeld et al. 2025). A distinct anterior–posterior pattern of volume asymmetry was observed, with anterior regions being larger on the right and posterior regions being larger on the left, akin to the human cerebral petalia (Zhao et al. 2022; Ocklenburg and Güntürkün 2024). However, these structural asymmetries did not align with established functional asymmetries in mice, indicating a more complex relationship that demands further investigation. This complexity challenges simple structure–function assumptions and calls for deeper inquiry into how anatomical asymmetries shape behavior (Silberfeld et al. 2025). In light of these findings, researchers must adopt more nuanced approaches to studying hemispheric roles in behavior, moving beyond assumptions of direct structure–function correspondence.

Previous analysis of 507 high‐resolution images from the Allen Mouse Brain Connectivity Project involving two mouse strains revealed that many regions display a density bias toward either the right or left hemisphere, with some areas showing a volume bias favoring the right hemisphere (Elkind et al. 2023). In particular, there is an increase in cell density, specifically in layer 2/3 of the left hemisphere, while no changes are observed in other layers (Elkind et al. 2023). These findings further emphasize the importance of accounting for regional‐ and cellular‐level asymmetries when constructing models of cognitive function and neurological disease.

## 3 Methodological Gaps and Technical Challenges

Despite growing awareness of hemispheric asymmetry in rodent neuroscience, there remains a notable lack of consistency in methodologies. A key challenge is the common practice of pooling data from both hemispheres for analysis, assuming that lateralized functions can be captured through averaged data. This approach overlooks critical structural and functional asymmetries unique to each hemisphere, potentially leading to an incomplete or inaccurate understanding of brain organization.

Pooling data across hemispheres can lead to biased or misleading conclusions, particularly in cases where one hemisphere exhibits stronger or more robust asymmetries than the other. When hemisphere‐specific effects are ignored, especially in research exploring lateralized cognitive functions, findings risk being misinterpreted or rendered invalid. Evidence suggests that hemispheric asymmetries play a central role in the brain's ability to process information efficiently, particularly in tasks that require complex cognitive functions such as language processing, emotion regulation, and sensory integration (Vallortigara and Rogers 2020; Ocklenburg and Mundorf 2022). Thus, failing to address hemispheric differences may result in inaccurate models of brain function that do not reflect the complexities of real‐world neural processes.

Moreover, methodological inconsistencies in hemisphere‐specific dissection further compromise reproducibility and validity. Rodent studies often neglect to isolate or analyze both hemispheres consistently, leading to variable sample sizes and representation. Some studies examine only one hemisphere in isolation, especially when researchers rely on simplified protocols to minimize experimental complexity. This practice may be convenient, but it risks overlooking critical hemisphere‐specific differences, particularly in regions with pronounced asymmetries, such as the amygdala, hippocampus, and prefrontal cortex. Oversimplified methods increase the risk of misinterpretation and limit the generalizability of findings to more nuanced models of brain function.

Additionally, the reliance on existing reference atlases, such as the Allen Mouse Brain Atlas, exacerbates these issues due to inconsistent hemisphere‐specific data inclusion (Grange et al. 2013, 2014; Oh et al. 2014; Eastwood et al. 2019; Wang et al. 2020). For example, some regions aggregate data from multiple subjects, mixing left and right hemispheres, while in other cases, data are available from only one hemisphere. These inconsistencies introduce potential biases and undermine result accuracy, especially in studies of lateralized brain regions or asymmetrical connectivity.

Even when hemisphere‐specific data are available, technical limitations in traditional imaging methods, such as MRI or histology, can hinder the detection of fine‐grained asymmetries in brain subregions. Recent advances, such as optogenetics, electrophysiology, and single‐cell RNA sequencing, offer promising avenues for more precise hemispheric investigations at cellular and molecular levels. However, these techniques are still emerging in the context of hemispheric asymmetry studies in rodents, with challenges in optimizing protocols. For instance, current electrophysiological and optogenetic approaches typically stimulate one hemisphere at a time or apply bilateral stimulation (Aslan et al. 2025; den Bakker et al. 2025), limiting direct hemispheric comparisons.

There is a pressing need for experimental protocols that allow independent or alternating stimulation and recording from both hemispheres within the same animal. This would enable researchers to precisely evaluate each hemisphere's contribution to behavior and neurophysiological processes, facilitating a more accurate assessment of lateralized functions. The absence of such protocols constitutes a critical gap in studying hemispheric asymmetry. A promising example is provided by Keary and Li (2025), who developed a dual‐hemisphere stimulation and recording setup in mice, enabling within‐subject comparisons of hemispheric contributions to sensorimotor integration (Keary and Li 2025). Their approach demonstrates the feasibility and importance of symmetrical experimental designs in asymmetry research.

Furthermore, the heterogeneity of rodent models presents another challenge for studying hemispheric asymmetry. Studies often rely on specific strains of mice or rats, which may differ in their inherent lateralization patterns. For example, strain‐specific differences in the size, shape, or functional connectivity of brain regions could confound results, especially when hemisphere‐specific patterns are generalized across different models (see Melone et al. 1984; Stieger et al. 2021; Elkind et al. 2023). Without standardized protocols, combined with strain‐related variability, findings across studies may remain inconsistent and difficult to interpret.

The lack of consideration for environmental factors that may influence brain lateralization in rodent models is another methodological limitation. Variables such as early life experiences, maternal care, and stress exposure can all shape hemispheric organization (de Kovel et al. 2019; Berretz et al. 2020; Mundorf et al. 2020; Mundorf and Ocklenburg 2023), yet they are often overlooked in animal studies investigating lateralization. Ignoring these factors risks a misunderstanding of the underlying neurobiological mechanisms that drive hemispheric asymmetries and their role in cognitive and emotional processes.

Finally, while large‐scale datasets, such as those from the Allen Mouse Brain Connectivity Project or other neuroimaging cohorts, hold promise for revealing lateralization patterns across multiple animals, these datasets are often limited by their cross‐sectional nature. Without longitudinal data, it is difficult to track the development of hemispheric asymmetries or to understand how lateralization evolves in response to environmental factors or neurological changes. Furthermore, cross‐sectional datasets may not fully capture the complex interplay between structure and function in lateralized brain regions, leading to an incomplete understanding of how asymmetries contribute to behavioral outcomes.

## 4 Emerging Tools for Investigating Hemispheric Specialization

One of the most promising advances in the study of hemispheric asymmetries is the application of genetic‐ and circuit‐level tools, which allow researchers to manipulate specific genes implicated in lateralization. CRISPR‐Cas9 gene editing, for example, enables the precise knockout or modification of genes (Asmamaw and Zawdie 2021) that may contribute to lateralized functions in the brain. This provides a way to study causal relationships between genetic variations and asymmetries in rodent models, an approach not feasible with traditional methods. By selectively manipulating genes involved in brain development, neural circuit formation, and hemispheric specialization, such as genes related to microtubules (Ocklenburg et al. 2025), researchers can uncover how these genetic factors contribute to observed hemispheric differences.

Additionally, viral vector–based gene delivery allows for hemisphere‐specific expression of light‐sensitive opsins (e.g., channelrhodopsin), enabling the optogenetic stimulation of hemisphere‐specific pathways. When combined with behavioral assays or neural recordings, this approach can reveal lateralized contributions to cognition, emotion, or sensorimotor control.

However, practical limitations remain. Independent or alternating hemisphere stimulation is rarely implemented due to surgical and technical complexity. Most optogenetic studies apply bilateral stimulation or focus on a single hemisphere, missing the opportunity to uncover lateralized dynamics. Similarly, electrophysiological techniques are rapidly advancing, with options for multi‐electrode arrays and silicon probes enabling fine‐grained recordings. Yet, synchronous bilateral recording within the same animal is still uncommon, limiting the ability to compare hemispheres directly under identical behavioral or physiological conditions. Single‐cell transcriptomics, spatially resolved gene expression mapping, and in situ sequencing further enrich the toolkit for exploring hemisphere‐specific molecular signatures. These methods, although technically demanding and resource‐intensive, have already revealed cell type–specific lateralization in cortical regions (Elkind et al. 2023). Such techniques hold significant potential for identifying molecular correlates of structural and functional asymmetries, particularly when combined with circuit‐level manipulation and in vivo imaging.

Despite their promise, these tools face real‐world constraints, including cost, labor demands, and feasibility within typical grant cycles, which may hinder their widespread adoption. Establishing standardized, cost‐effective protocols for bilateral experimentation will be a crucial next step in making hemispheric research more accessible and scalable. This Spotlight article aims to underscore the practical and conceptual importance of integrating hemispheric considerations in experimental design and may serve as a useful reference when justifying protocol adjustments or funding applications.

## 5 Recommendations for Future Research Practice

**FIGURE 1:**
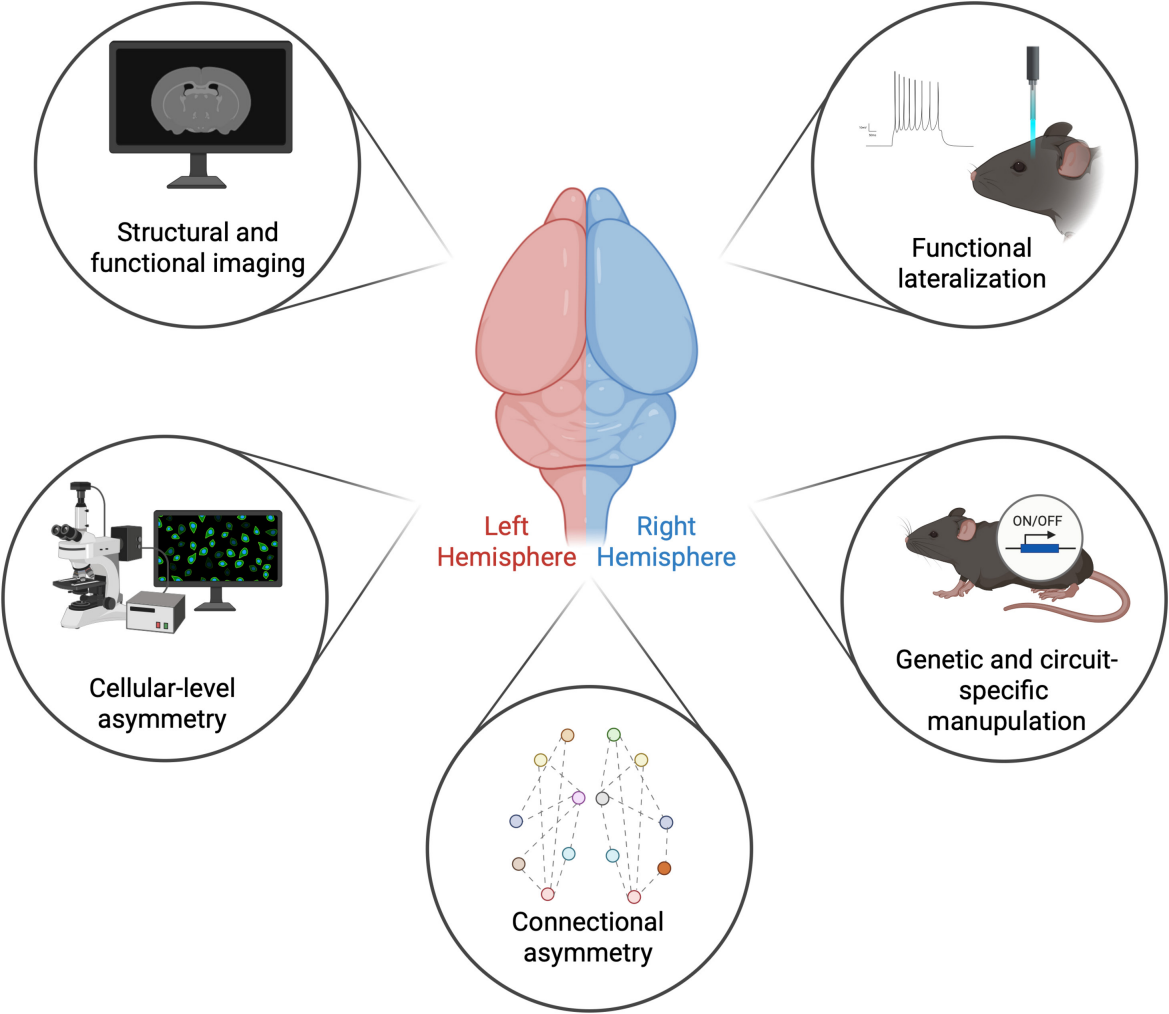
Practical strategies for advancing hemispheric asymmetry research in rodents. The figure illustrates key methodological approaches for studying hemispheric asymmetry in rodent neuroscience. At the center is a schematic of a rodent brain, with the left hemisphere shown in red and the right in blue, underscoring the importance of analyzing each hemisphere independently. The surrounding labels represent major domains of asymmetry research: *structural and functional imaging* (top left), *cellular‐level asymmetry* (bottom left), *Connectional asymmetry* (bottom center), *Functional lateralization* (top right), and *genetic‐ and circuit‐specific manipulation* (bottom right). Each domain highlights specific methodological needs. Imaging studies should account for interhemispheric variability to avoid averaging out meaningful differences. Electrophysiological and optogenetic experiments benefit from parallel or alternating hemisphere recordings to capture lateralized function. Cellular and molecular analyses must maintain hemisphere‐specific resolution to detect subtle but significant asymmetries. Genetic‐ and circuit‐level interventions should be designed with potential hemisphere‐dependent effects in mind. Applying these strategies increases accuracy, reproducibility, and translational relevance by ensuring that lateralized patterns are appropriately captured rather than obscured. Created with BioRender.com.

To advance the study of hemispheric asymmetry in rodent neuroscience, we recommend that researchers adopt the following practical strategies aimed at improving validity, reproducibility, and translational relevance. These are visually summarized in Figure 1, which highlights five key methodological domains essential to asymmetry research: (1) structural and functional imaging, (2) cellular‐level asymmetry, (3) connectional asymmetry, (4) functional lateralization, and (5) genetic‐ and circuit‐specific manipulation. Building on the methodological domains highlighted in Figure 1, the following recommendations outline concrete steps researchers can take to improve the study of hemispheric asymmetries in rodents:

- **Separate Hemispheric Dissection**: Consistently dissect and analyze both hemispheres separately to avoid pooling data that may obscure asymmetries. Use consistent protocols for dissection to ensure comparability.
- **Use Hemisphere‐Specific Analyses**: Perform analyses for each hemisphere individually, ensuring that structural and functional differences are captured accurately.
- **Refine Optogenetics Protocols:** Develop protocols for independent or alternating hemisphere stimulation in the same animal, allowing for clearer evaluation of each hemisphere's role in behavior and brain activity.
- **Electrophysiological Recordings**: Record from both hemispheres simultaneously or in separate conditions to capture hemisphere‐specific neural activity. Use multi‐site recordings to explore cross‐hemisphere interactions when needed.
- **Increase Sample Sizes**: Ensure sufficient sample sizes for each hemisphere to increase statistical power and reduce bias, especially when studying handedness or lateralized traits.
- **Track Developmental and Environmental Factors**: Consider early life experiences, stress, or environmental factors that may influence hemispheric asymmetries. Where feasible, track these variables longitudinally to observe developmental trajectories.
- **Leverage Advanced Imaging and Resources**: Use high‐resolution imaging (e.g., 2‐photon microscopy) and advocate for hemisphere‐specific data in reference atlases like the Allen Mouse Brain Atlas for more accurate interpretation.
- **Standardize Protocols**: Establish standardized experimental protocols for hemisphere‐specific research, ensuring consistency across studies and better comparison of results.
- **Integrate Emerging Technologies**: Combine optogenetics, electrophysiology, and single‐cell sequencing to study hemispheric differences at both the circuit and molecular levels. Such multi‐modal approaches offer more comprehensive insights into lateralized brain functions.

Several recent high‐profile studies illustrate the methodological challenges that continue to obscure hemispheric asymmetries in rodent neuroscience, despite growing awareness of their importance. For instance, Zhang et al. (2023) and Van Deusen et al. (2025) generated comprehensive whole‐brain atlases but pooled data across hemispheres and individuals, making it difficult to detect lateralized cellular or transcriptional patterns. To better capture asymmetries, it is recommended that future atlas‐building efforts include bilateral sampling from the same animal and explicitly annotate the hemisphere of origin.

In Jain et al. (2024), dendritic plasticity was examined in a single hemisphere, leaving the potential for lateralized signaling unexplored. For a more accurate assessment of asymmetrical mechanisms, it is advisable to implement alternating or independent hemisphere‐specific stimulation and imaging protocols within the same subject.

Studies such as Kremer et al. (2024) and Sayar‐Atasoy et al. (2024) employed advanced molecular and circuit‐level techniques, yet did not distinguish between hemispheres in their analyses. In such cases, incorporating hemisphere as a factor in experimental design and statistical analysis is essential for detecting potential lateralization.

These examples underscore the importance of refining experimental approaches. For improved detection of hemispheric asymmetries, it is recommended to consistently perform hemisphere‐specific dissections, record bilaterally when feasible, and report hemisphere of origin in all datasets. Such practices will increase the precision and reproducibility of findings in rodent neuroscience.

## 6 Conclusion

Recent large‐scale studies have demonstrated the crucial importance of considering hemispheric asymmetries to advance our understanding of rodent neuroanatomy and neurophysiology. These findings underscore the need to prioritize hemisphere‐specific analyses in order to fully capture the complexity of brain function. With rapid advancements in neuroscience techniques, such as optogenetics, high‐resolution imaging, and single‐cell profiling, rodent models are now better positioned than ever to support more nuanced investigations of lateralized brain processes.

However, current methodologies often fall short. The pooling of hemispheric data, inconsistencies in dissection protocols, and dependence on incomplete reference atlases undermine both the validity and generalizability of results. Furthermore, the limited resolution of traditional imaging tools and the lack of attention to strain‐specific or environmental variables complicate the interpretation of asymmetry‐related findings.

To overcome these barriers, future studies must adopt more rigorous and standardized approaches. This includes hemisphere‐specific protocols, high‐resolution and longitudinal imaging, and multi‐strain experimental designs. Expanded use of genetic tools, bilateral electrophysiology, and optogenetics and hemisphere‐aware data repositories will also be essential.

By systematically addressing these methodological gaps, researchers can uncover novel insights into the neurobiological mechanisms underlying psychiatric and neurological disorders, many of which involve disrupted lateralization. A shift toward more precise, hemisphere‐conscious research will not only deepen our understanding of the brain's asymmetrical organization but also enhance the translational relevance of rodent models in neuroscience.

## 7 Outlook

To further advance the field of hemispheric asymmetry in rodent neuroscience, several key areas need further exploration. First, it remains unclear to what extent the asymmetries observed in rodents mirror those in humans, or whether interspecies differences limit translational relevance. Clarifying these relationships is essential for validating rodent models as tools for understanding human brain lateralization.

Second, methodological inconsistencies, particularly in hemispheric dissection, complicate cross‐study comparisons. While some studies analyze only one hemisphere, others include both, without clear rationale, increasing the risk of biased or incomplete findings. Establishing standardized dissection and analysis protocols will be crucial for improving reproducibility.

Finally, the widespread simplification of data collection, such as omitting bilateral sampling or neglecting hemisphere‐specific analysis, may obscure critical lateralization patterns, particularly in domains of affective regulation, memory, and reward processing. Future research should adopt more comprehensive, hemisphere‐aware methodologies to better reflect the underlying organization of the brain.

Prioritizing such approaches will not only refine our understanding of rodent neurobiology but also strengthen the translational potential of these models in investigating psychiatric and neurological disorders.

## Author Contributions

Both authors contributed equally.

## Conflicts of Interest

The authors declare no competing interests.

## Peer Review

The peer review history for this article is available at https://www.webofscience.com/api/gateway/wos/peer‐review/10.1111/ejn.70249.

## Data Availability

The authors have nothing to report.
